# Impact of Stress on Periodontal Health: Literature Revision

**DOI:** 10.3390/healthcare11101516

**Published:** 2023-05-22

**Authors:** Denise Corridore, Matteo Saccucci, Giulia Zumbo, Erika Fontana, Luca Lamazza, Claudio Stamegna, Gabriele Di Carlo, Iole Vozza, Fabrizio Guerra

**Affiliations:** Department of Oral and Maxillo-Facial Sciences, Sapienza University of Rome, 00185 Rome, Italy; denise.corridore@uniroma1.it (D.C.); matteo.saccucci@uniroma1.it (M.S.); fontana.1850673@studenti.uniroma1.it (E.F.); luca.lamazza@uniroma1.it (L.L.); claudio.99.stamegna@gmail.com (C.S.); gabriele.dicarlo@uniroma1.it (G.D.C.); iole.vozza@uniroma1.it (I.V.);

**Keywords:** stress, periodontal health, literature revision, impact

## Abstract

Stress is a physiological response of the body to stressful life events but may not be when the individual is persistently exposed to the stress trigger, and it negatively affects certain physiological functions, thus triggering psychosomatic diseases. In literature, chronic stress and inadequate coping strategies are found to mediate the risk and development of periodontitis; mechanisms have therefore been proposed to explain the effects of stress on the periodontium. Since stress is a prevalent problem in modern life and given the importance of maintaining oral health, the present literature review aimed to estimate the association between stress and periodontal disease. The research question adopted is the following: “Are psychological stress and periodontal disease related?” The search was conducted in August 2022 and limited to articles in electronic databases from 2017 to 2022 in English, excluding reviews and literature reviews. From the electronic databases, a total of 532 articles were identified and became 306 after reviews and duplicates were removed. An additional bibliographic search was conducted through the same electronic databases, controlled terms and keywords including only systematic reviews, which were previously excluded. Through the bibliography cited in the systematic reviews, an additional 18 articles were identified, with a new total of 324. As a result of reading the title and abstract of these 324 articles, an additional 295 were excluded. Reading the full text of the remaining 29 studies, 2 articles were excluded due to non-adherence to the eligibility criteria. The remaining 27 results were included in our literature review. It has been suggested in the literature that adverse socioeconomic conditions elicit a stress response, which can trigger periodontal inflammation. Most of the 27 articles included in the study confirm and demonstrate a positive association between psychological stress and periodontal disease. Numerous studies have shown the mechanisms through which chronic stress negatively affects periodontal tissues. Therefore, in the light of the results obtained from this review, it is important that oral health professionals, also for general health purposes, consider stress factors among the risk factors of periodontal disease, its severity and decreased efficacy of treatments. It is therefore advisable to act preventively through the interception of chronic stress.

## 1. Introduction

Stress is defined as a physical, emotional, or mental response to stressful life events; it is a physiological response of the body but may not be when the individual is persistently exposed to the stress trigger. Additionally, it negatively affects certain physiological functions, thus triggering psychosomatic diseases [1].

Nowadays, the term stress is used with extreme frequency to describe recurring negative experiences related to everything from everyday inconveniences, relationship problems and work pressures to health problems and debilitating phobias.

Exposure to stressors by the body results in the triggering of a series of physiological changes. Stress, depending on its duration, can be distinguished as acute or chronic. These responses set in quickly and just as quickly decay, with a return to homeostasis.

In the context of periodontal diseases, (i.e., diseases compromising the supporting apparatus of the tooth, which includes gingival tissue, alveolar bone, cementum and periodontal ligament) [2], severe periodontitis is the sixth-most common chronic noncommunicable disease in the world, with a preponderance of 11.2 percent of the world population, with peak incidence around age 40 [3].

There are two different forms of periodontal disease: gingivitis and periodontitis.

Host susceptibility to periodontal disease in terms of extent, severity, progression and response to therapy can be modified by external and internal components, which can be classified into modifiable risk factors and non-modifiable risk factors. The factors that are modifiable, as their elimination or control is critical in the management of periodontal disease, are plaque/tartar, overflowing restorations, partial dentures and dental malposition, while systemic factors include excessive alcohol consumption, smoking, stress, medications and certain medical conditions such as poorly controlled diabetes, obesity, osteoporosis, low dietary calcium and vitamin D [4,5].

Other systemic factors including certain clinical conditions such as unstable diabetes and leukemia, AIDS, Crohn’s disease and genetic factors such as positive family history of periodontal disease cannot be modified, and are therefore defined as non-modifiable [4,5].

Studies in the literature indicate that chronic stress and inadequate coping strategies may mediate the risk and development of periodontitis; mechanisms have therefore been proposed to explain the effects of stress on the periodontium. Stress would appear to indirectly influence periodontal health by inducing changes in unhealthy coping behaviors such as smoking, excessive alcohol consumption and illicit drug use, improper diet, neglect of oral hygiene and poor compliance with dental care; it would also appear to influence periodontal health through direct biological impact, mediated through alteration of saliva, changes in gingival blood circulation and by influencing the host immune response [6].

The role of the influence of psychosocial disturbances on the periodontium is not new; on the contrary, stress for many decades has been reported as a risk factor for necrotizing ulcerative gingivitis (NUG), a condition known by the term “trench mouth” associated with bacterial infection and characterized by papillary necrosis, pain and gingival bleeding [7]. This association has recently been confirmed by several studies, where during the COVID-19 pandemic, a period characterized by increased levels of stress compared to the pre-pandemic period, an increased number of necrotizing periodontal lesions were found [8].

A patient’s mental health status influences dental hygiene to some extent. Poor oral hygiene due to stress can promote plaque accumulation and over time the occurrence of gingivitis and periodontitis [6].

Several articles in the literature state that dental and medical students are subject to higher stresses. The components post-underestimation had a significant increase during undergraduate exams, compared with post-exams. In conclusion, academic stress has a negative effect on plaque levels and gingival status [9,10,11].

Furthermore, saliva performs numerous functions. Among these, it plays a key role in chewing, swallowing, phonation and maintaining oral health. In the oral cavity, it performs the function of antibacterial, antifungal, antiviral, furthermore it cleanses and lubricates the dental elements and oral mucosa; likewise, it modulates demineralization and possesses a buffering capacity that promotes the maintenance of neutral pH [12]. Salivary glands are strictly under the control of the parasympathetic system [13], unlike under stress conditions, where sympathetic stimulation predominates, resulting in reduced salivary flow [14], which can negatively affect periodontal health [6]. When the protective action of saliva is lacking, especially if persistently, mucosal changes, increased incidence of dental caries and opportunistic infections occur [15,16].

Since stress is a prevalent problem in modern life and given the importance of maintaining oral health, the present work aimed to estimate the association between stress and periodontal disease through a literature review investigating the main relations highlighted in the literature regarding this topic.

## 2. Materials and Methods

This systematic review was conducted following the criteria of the “Preferred Reporting Items for Systematic Reviews and Meta-Analyses” (PRISMA) Statement (Liberati et al., 2009).

The PECO (Population, Exposure, Comparator, and Outcome) model was used for formulating the scientific question and as guidance of the inclusion and exclusion criteria of this review. This acronym represents the characteristics of the patient (P), the exposure (E), the comparator (C), and the outcome (O) of the eligible question. The research question adopted is as follows: “Are psychological stress and periodontal disease related?”

- Population (P): human subjects with periodontal disease;
- Exposure (E): psychological stress assessed by specialized questionnaires or biological biomarkers;
- Comparison (C): periodontally healthy subjects not exposed to psychological stress;
- Outcome/Results (O): association between psychological stress and the presence of periodontal disease.

The search was conducted in August 2022 and limited to articles in electronic databases from 2017 to 2022 in English, excluding reviews and literature reviews.

Bibliographic research was conducted by two independent operators through the electronic databases—Pubmed, Scopus and Web of Science—using combinations of selected terms and different keywords, depending on the search mode allowed by each electronic database as shown in Table 1. Additional research was conducted through the bibliography of systematic reviews with the same topic.

The research began in July 2022, continued in August and ended on 6 September 2022.

Study selection was performed by two independent operators following the process of Identification, Screening, Eligibility and Inclusion of studies.

Articles were saved in the Zotero software (version 6.0.11, 18 July 2022), where duplicates were removed. A preliminary screening was then performed by two independent operators, which involved reading the titles and abstracts of the articles, in order to remove the studies not relevant to the purpose of this review.

The eligibility process was carried out by reviewing and analyzing the full texts for further inclusion or exclusion. Access to the full texts of the articles was obtained through the “Nilde” (Network Interlibrary Document Exchange) system, in collaboration with Sapienza University of Rome.

The papers included in the study were randomized controlled trials (RCTs) and con-trolled clinical trials (CCTs), cohort studies, case–control studies and transversal studies.

The excluded studies were literature reviews, articles that were not in English, articles whose full text was not available and studies not carried out on humans.

**Figure 1:**
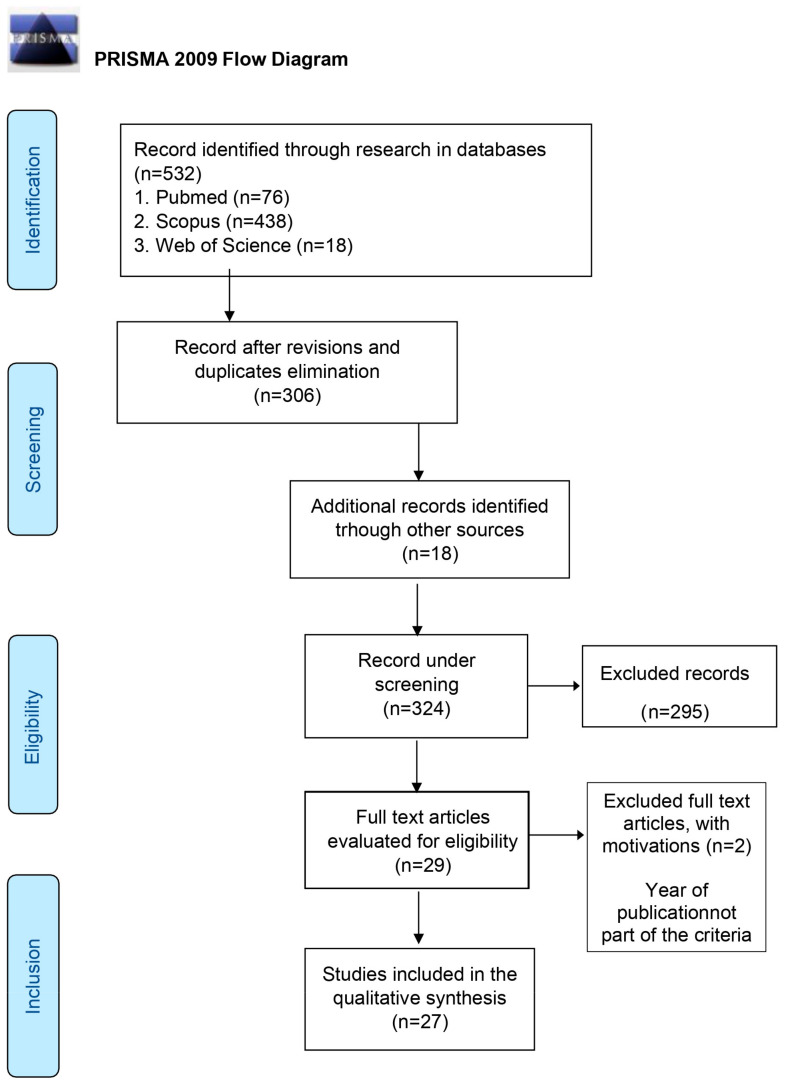
Prisma Flow Diagram.

The entire selection process was schematized in the PRISMA Flow Diagram (Figure 1).

Data collection was carried out by reading the full texts of the included studies. with related and subsequent manual entry into the tables, using the Microsoft Excel spreadsheet (Table 2 in result section).

The following information was extracted from the studies: author; year of publication; country; study type; sample size/(case/control numbers); gender; age group/middle age; inclusion and exclusion criteria; diagnosis of periodontal disease; instruments used to measure stress; and conclusions.

The methodological quality of the included studies was performed by two independent practitioners using “The modified JADAD scale” (Oremus et al., 2001). The modified scale includes nine criteria or items to which scores are assigned. The score for each study ranges from 0 (the lowest quality) to 8 (the highest quality), where 0 represents the highest risk of bias and 8 represents the lowest risk of bias. Studies with scores of 7 and 8 were classified as low risk, those with scores of 4, 5 and 6 were classified as moderate risk and studies with scores less than or equal to 3 as high risk. Studies scoring less than 4 were considered low quality, studies scoring 4 or more (with a maximum of 6), were considered medium quality and studies scoring more than 6 were considered high quality.

The quality of the selected studies was assessed using the modified version of the Newcastle–Ottawa scale for cross-sectional studies by Wells et al. [17].

Study quality was rated on a scale from 0 (high risk of bias) to 8 (low risk of bias). The scale assessed the parameters of sample selection, comparability and outcome/exposure. Studies that showed a summary score greater than the median value were considered to be at low risk of bias. Each parameter was awarded 1 point. Any disagreements among the reviewers in assessing study quality were resolved by consensus or by consulting a third reviewer.

To reduce the bias and time spent in the study selection process, a Cohen’s kappa statistic system was used in the process of selecting studies. An iterative process based on the use of this statistic during which the criteria were refined until obtaining almost perfect agreement (k > 0.85). At this point, the two researchers interpret the selection criteria in the same way; therefore, the bias is reduced.

## 3. Results

From the electronic databases Pubmed (n = 76), Scopus (n = 438) and Web of Science (n = 18), a total of 532 articles were identified, 306 after duplicates were removed. In addition, an additional bibliographic search was conducted through the same electronic databases, controlled terms and keywords including only systematic reviews, which were previously excluded. Through the bibliography of the latter, an additional 18 articles were identified, with a total of 324.

As a result of reading the title and abstract of the latter, an additional 295 were excluded. Reading the full text of the remaining 29 studies resulted in the exclusion of 2 articles due to non-adherence to the eligibility criteria. The remaining 27 results were included in the literature review. The process was schematized in the PRISMA Flow Diagram (Figure 1).

The characteristics of the included studies are shown in Table 2. All included studies were published between 2017 and 2022. These were conducted in several countries, with a high percentage conducted in India (Varshini and Rajasekar, 2020; Sudhakar et al., 2017; Obulareddy et al., 2018; Bawankar et al., 2018; Fenol et al., 2017; Rahate et al., 2021; Rajhans et al., 2017; Ramesh et al., 2018); the remaining were conducted in Japan (Islam et al., 2019; Maruyama et al., 2022), Turkey (Develioglu et al., 2020; Yarkac et al., 2018), France (Dubar et al., 2020; Petit et al., 2021; Petit et al., 2021), China (Deng et al., 2021; Zhang et al., 2021), Iran (Karimi et al., 2017; Naghsh et al., 2019), Austria (Haririan et al., 2018), Brazil (Coelho et al., 2020), Sri Lanka (Wellappulli et al., 2019), Saudi Arabia (Tanveer et al., 2021), Nigeria (Folayan et al., 2021), Indonesia (Wijayaa et al., 2020), United Arab Emirates (Khalil et al., 2020), and Canada (Gomaa et al., 2020) [1,16,18,19,20,21,22,23,24,25,26,27,28,29,30,31,32,33,34,35,36,37,38,39,40,41,42].The number of participants involved in each study ranged from 27 to 1087 of both sexes, with an age range of 10 to 74 years.

Periodontal disease was diagnosed by radiographic and clinical criteria. The latter were performed through periodontal measurements of several parameters such as clinical at-tachment level (CAL), plaque index (PI), probing depth (PD), bleeding on probing (BOP), plaque control record (PCR), gingival index (GI), tooth mobility (DM), papillary bleeding index (PBI), periodontal surface inflamed area (PISA), oral polymorphonuclear leukocytes (OPMN), oral inflammation (OIL), oral hygiene index (OHI), simplified oral hygiene index (OHI-S), community periodontal index (CPI), papillary, marginal and adherent gingival index (PMA), and gingival recession (REC). Stress was measured by administering several questionnaires, among them the Depression, Anxiety and Stress Scales (DAS), Perceived Stress Scale (PSS), Hospital Anxiety and Depression Scale (HADS), Percived Stress Questionnaire (PSQ), State and Trait Anxiety Index (STAI), Symptom Checklist 90 (SCL-90), General Health Questionnaire (GHQ-30), Patient Health Questionnaire 9 (PHQ-9), Beck De-pression Inventory (BDI), Scale for Assessing Academic Stress (SAAS), Toulouse Coping Scale (TCS), and Self-Report Psychopathy Scales (SRP). Salivary and serum samples were also taken for the assessment of cortisol, IgA, ghrelin, chromogranin-A, salivary al-pha-Amylase, substance P (SP), calcitonin gene-related peptide (CGRP), vasoactive intes-tinal peptide (VIP), neuropeptide Y (NPY), Adrenomedullin (ADM).

**Table 2:** The characteristics of the studies included in the research.

Author, Year, Nation	Type of Study	Total Dimension of Sample (n of Case/Control)	Gender, -Age Range, Mean Age	Inclusion/Exclusion Criteria (C/EC)	Diagnosis of Periodontal Disease	Stress Measurement Tools	Conclusions
Wellappulli et al., 2019 Sri Lanka [38]	Case–control	694 CP, 706 healthy	680 F 720 M 30–60	IC: 30–60 y.o. adults. Sri Lanka EC: n of teeth < 20; pregnant women; disability.	PD, CAL	GHQ-30	Psychological distress is an independent risk factor for chronic periodontitis (CP) in the Sri Lankan population.
Varshini and Rajasekar 2020 India [16]	Clinical Observational	100 dentistry students	NR NR	NR	Presence or absence of PD > 3 mm and loss of CAL	DAS	Dental students with severe levels of depression, anxiety and stress presented greater pocket depth and CAL.
Sudhakar et al., 2017 India [18]	Cohort study	30 G1: 15 CP G2: 15 CP stress induced	Both sexes, 30–45	EC: systemic diseases; antibiotic assumption, antioxidants; smokers.	BOP, PI, CAL, xray exam, ROM seric level measurement	Salivary and serological cortisol	The role of stress in the progression of periodontal disease was associated with increasing cortisol and ROM levels.
Islam et al., 2019 Japan [25]	Transversal	738 Japanese workers	92 F 646 M 19–65 40.7 ± 10.5	EC: incomplete questionnaire.	Objective method: redness and/or swelling of the gingiva, with gingival recession and/or tooth mobility	Job stress and coping style questionnaire: Co-Labo57+	Low coping style in relation to high job stress was significantly associated with periodontitis.
Develioglu et al., 2020 Turkey [27]	Transversal	80 G1: 26 mild chronic periodontitis; G2: 39 moderate chronic periodontitis; G3: 15 severe chronic periodontitis	37 F 43 M Group 1. 38.23 ± 3.55 (35–49) Group 2: 42.66 ± 7.65 (35–62) Group 3: 55.26 ± 6.94, (41–68)	IC: no dentures; not lactating. EC: diabetes; heart disease; allergies; smokers and alcohol users; periodontal treatment in the previous 6 months; anti-inflammatory or antioxidant drugs; antibiotics	PI, GI, CAL, PD, radiographic exam	STAI 1 and 2; pancreatic chromogranin A/statin, amylase alpha 1, beta-endorphin and salivary cortisol	Chronic periodontitis increased as salivary cortisol levels increased, and there was no correlation with the levels of other stress markers in saliva.
Obulareddy et al., 2018 India [19]	Transversal	92	51 F 41 M NR	IC: age ≥ 30; muber of dental elements > 20; absence of systemic diseases, drug absumption and history of periodontal tratment over the last 6 months. EC: pregnant and nursing women	PD, CAL, BOP, PI	DASS-21, Salivar cortisol	Cortisol was associated with both chronic periodontitis (CP) and psychological stress. Cortisol levels were higher in subjects with CP associated with stress, compared with subjects with the presence of CP or stress alone.
Haririan et al., 2017 Austria [36]	Case-control	56; 21 AgP,35 CP 44 healthy	20 F 36 M 44; 21–64	Control group IC: Healthy individuals; no PD ≥ 4 mm; no radiographic bone loss.	PD, CAL.	PSQ; Weariness scale; Warning Signals; SVF78; serum and salivary cortisol; serum levels of BDNF, SP, CGRP, VIP, NPY, ADM.	NPY and salivary VIP could be related to periodontal disease, regardless of stress levels or coping strategies. Salivary and serum cortisol levels did not differ between health and disease states.
Coelho et al., 2020 Brazil [37]	Transversal	621	300 F 321 M 59.42 ± 10.91 (± SD)	IC: n of teeth > 4; not pregnant; no cancer or HIV-AIDS; no anti-inflammatory drugs; no periodontal treatment in the 6 months prior to study. EC: history of stroke; percutaneous coronary revascularization in the previous 6 months or surgical revascularization in the 2 months prior to the study.	PD, REC, CAL, BOP, PI e number of teeth	PSS	Positive association between stress exposure and presence of periodontitis.
Maruyama et al., 2022 Japan [26]	Cohort study-prospective	40	NR NR	IC: age ≥ 20 years. EC: systemic disease; periodontal treatment within 3 months; smokers; antibacterial, anti-inflammatory, or antiallergic medications within the past 2 weeks.	Oral status examinations-nato at T0 and follow-up. PD, BOP, PISA, PCR	Questionnaires completed at T0 and follow-up by self-reported questionnaires: PHQ-9, BDI and MIMOSYS to assess mental health status.	The change in psychological stress over 14 days was significantly correlated with changes in PD and the degree of periodontal inflammation.
Dubar et al., 2020 France [29]	Case-control	60; 30 patients with periodontitis 30 patients without periodontitis	34 F 26 M. Cases (38–65, mean age 55.0) Control (22–70, mean age 55.0) 34 F 26 M. Cases (38–65, mean age 55.0) Control (22–70, mean age 55.0)	IC: Case group: patients aged ≥ 18; moderate to severe chronic periodontitis; with at least two periodontal pockets (PD ≥ 5 mm) and one healthy site (PD ≤ 3 mm); without pathology; no history of taking antimicrobials, anti-inflammatory drugs; not pregnant; no periodontal treatment in the previous 6 months. -Control group: absence of periodontal disease; in good health; not pregnant.	Collection of parameters before and after nonsurgical periodontal treatment: PD, CAL, BOP, PI, GI, tooth mobility, radiographic examination, microbiological sample collection through crevicular fluid (GCF)	STAI-Y, PSS, salivary cortisol	Stress and anxiety in periodontal patients, appear to be associated with different bacterial colonization after SRP of Socransky orange or red complexes between stressed/anxious and non-stressed/anxious patients. Salivary cortisol concentrations would appear to be related to pocket depth, but not to self-reported stress/anxiety scores.
Deng et al., 2021 China [32]	Transversal	460	NR 45–46	IC: age ≥ 18 years with a diagnosis of periodontitis. EC: inability to understand and independently complete questionnaires; aggressive systemic diseases.	CAL, PD	HADS, PSS-10	The present study showed an increased prevalence of anxiety, depression, manifestations of distress and moderate to high stress among patients with periodontitis.
Bawankar et al., 2018 India [20]	Observational	75 G1: 25 pcs periodontally healthy G2: 25 pcs non-smokers with moderate to severe untreated CP G3: 25 pcs smokers with moderate to severe untreated CP	35 F 40 M 30–65	IC: G1: periodontally healthy pcs with no history of smoking. G2: pcs without smoking history with severe untreated CP, PPD ≥ 5 mm and CAL ≥ 5 mm (30% affected teeth), radiographic alveolar bone loss. G3: pcs with smoking history (at least 10 cigarettes per day over the last 3 years). And Severe untreated CP, PPD ≥ 5 mm and CAL ≥ 5 mm (30% of teeth affected), radiographic alveolar bone loss.	PD, CAL, PI, GI, PBI	Serum and salivary cortisol, IL-1	Strong correlation between emotional stress, smoking and CP. Smoking patients with CP show higher salivary cortisol, serum, IL-1, and stress levels than nonsmokers with CP, thus showing increased risk and severity of periodontal disease.
Tanveer et al., 2021 Saudi Arabia [39]	Transversal	385	F 15–30, M 31–45	IC: age range 15–45 years. EC: absence of consent; pregnant, with history of diabetes mellitus; dementia and hypodontia/anodontia.	CPI, PD, only the central incisors and first and second perma-nent molars were recorded.	PPS	Significant association between psychosocial stress and periodontitis among socially disadvantaged women residing in group homes.
Folayan et al., 2021 Nigeria [40]	Transversal	1.087	478 F 609 M 10–19	EC: mental problems and critically ill.	GI	PHQ-9	Mild depression was associated with higher probabilities of moderate/severe gingivitis in the study population and was a modifying factor for the association with plaque accumulation and refined carbohydrate consumption.
Naghsh et al., 2019 Iran [35]	Analytical-Transversal	90: 45 CP, 45 N-CP	CP: 9 F, 36 M N-CP: 40 F, 5 M 20–55 CP: 37.1 ± 9.8 y.o. (range: 23–55 y.) N-CP: 34.8 ± 10.7 y.o. (range: 20–55 y)	IC: at least 5 natural teeth. EC: systemic diseases; or chronic immunosuppressant drugs, antidepressants, diuretics or psychoactive drugs, antihistamines, tricyclic antidepressants; smokers or alcohol users; pregnant or lactating; stressed patients undergoing treatment; orthodontic therapy; symptoms of acute disease or pulpal pain at the time of the study; history of periodontal treatment and antibiotic intake in the past 6 months.	PD, PI, BOP	STAI, salivary cortisol	Increased salivary cortisol levels in patients with CP. Therefore, the likelihood of occurrence of periodontitis is higher in subjects with increased cortisol levels.
Fenol et al., 2017 India [21]	Transversal	70 G1: PD > 4 mm e < 6 mm G2: PD ≥ 6 mm G3: PD ≤ 3 mm	M 25–60 38.56 ± 10.878	IC: n.teeth > 20. EC: corticosteroids or immunosuppressive drugs; Addison’s disease or Cushing’s syndrome; smokers; systemic diseases; history of psychiatric disorder; female sex; history of periodontal therapy in the past 6 months.	PD, CAL, OHI-S, GI.	Salivary cortisol levels, DASS	There is a positive relationship between stress and periodontal disease.
Karimi et al., 2017 Iran [34]	Case-control	30 G1: 15 with periodontitis G2: 15 without periodontitis	15 F 15 M 42–44	IC: G1: no periodontal disease; G2: CAL ≥3 mm and BOP in upper teeth.	PD, CAL, PI, GI, BOP	DASS-42, IgA salivary	A total of 87% of subjects with periodontal disease were depressed compared with 60% without periodontal disease and depression.
Zhang et al., 2021 China [33]	Randomized, double-blind, prospective	600 G1: 200 individuals with CP, history of smohaing and depression; G2: 200 with CP, no history of smoking and depression; G3: 200 individuals parodontalmente sani	300 F 300 M 20–50	EC: systemic diseases; drug intake; benign pituitary tumors including adenomas; cancerous pituitary tumors; benign and malignant adrenal gland tumors; Cushing syndrome, corticosteroids to treat asthma, arthritis, and some cancers; pregnant women.	GI, PI, PD, CAL, BOP, dental mobility, radiographic evidence	SCL-90, salivary cortisol	Strong relationship between depression, smoking history and CP. Smokers with CP show significantly higher cortisol levels in saliva than in serum. Subjects with elevated cortisol levels are at increased risk for periodontitis.
Wijayaa et al., 2020 Indonesia [41]	Transversal	57 students	NR NR	IC: non-smokers for more than 6 months; no history of pregnancy, chronic diseases of the immune system, no use of antibiotics in the last 4 weeks; no use of immunomodulatory drugs, anti-inflammatory drugs, sodium antagonists and anticonvulsants; and no odotoiatric/orthodontic treatment.	Measurement of salivary and plaque flow rate (O’Leary)	SAAS	Academic stress in-fluences salivary flow rate but not directly on plaque score. A decrease in the salivary flow rate causes an increase in plaque score.
Petit et al., 2021 France [30]	Prospective	54	56%F 51,2 y.o	IC: age > 18 years; diagnosis of severe chronic periodontitis; CAL > 4 mm; n. of teeth > 15 with at least 5% of sites with PPD > 5 mm and radiographic bone loss. EC: systemic diseases; history of taking anti-inflammatory, psychotropic and antibiotic drugs in the past 6 months; pregnancy; orthodontic therapy; history of periodontal treatment in the past 6 months.	PD, CAL, PI, and BOP recorded at T0 before SRP, at T3 and T6	STAI, BDI	Depression and anxiety have been shown to be a risk factor of worsening SRP outcomes.
Petit et al., 2021 France [31]	Prospective	71 at T0 58 at T3 54 at T6	T0: 40 F, 31 M T3: 32 F, 26 M T6: 30 F, 24 M 29–74 mean age 51.3	IC: age > 18 years; n. of teeth > 15; diagnosis of severe chronic parodontitis; with at least 5% of sites with PD > 5 mm and radiographic bone loss. EC: systemic diseases; history of taking anti-inflammatory drugs, psychotropic drugs, and antibiotics in the past 6 months; pregnancy; orthodontic therapy; history of periodontal treatment in the past 6 months.	PI, BOP, PD, CAL (collected at T0, before SRP and at 3 months) Complete periodontal examination performed at 6 months	DASS-42, TCS, At T0 and T6 from the SRP, plasma levels of cortisol and chromogranin-A were collected	Patients with increased stress, anxiety and depression scores, who use negative coping strategies, show worse SRP scores.
Khalil et al., 2020 United Arab Emirates [1]	Comparative-observational	150	56 F 94 M 20–40	IC: male and female patients attending the dental clinics of Ajman University; age between 20 and 65; any nationality. EC: people not attending Ajman University dental clinics; age below 20 or above 65.	NR	Questionnaire adapted from Barreca and Hepler (2000)	Stress-related oral manifestations. Among the different manifestations, chronic periodontal disease had an incidence of 24%, with a greater impact in older and male patients.
Gomaa et al., 2020 Canada [42]	Transversal	102	52% di F 20–59	EC: chronic, autoimmune or inflammatory diseases; history of taking corticosteroid, antibiotic, probiotic or prescription drugs; hair treatments/coloring; hair length < 3 cm; dental procedures and periodontal treatment within the past 3 months.	OPMN, OIL, PD, BOP, CAL	PSS, FSS, hair cortisol concentration, salivary alpha-Amylase	Psychosocial stress may contribute to pro-inflammatory immunity, implicated in the pathobiology of periodontal disease.
Rahate et al., 2021 India [22]	Transversal	90 G1: periodontally healthy pcs G2: pcs diagnosed with stage III periodontitis and non-smokers G3: pcs diagnosed with stage III periodontitis and smokers	34 F 56 M G1: 18 F, 12 M G2: 14 F, 16 M G3: 5 F, 25 M 30–65 G1: 49.03 G2: 51.93 G3: 52.23	IC: G1: healthy, non-fumatorial pcs. G2: non-fumatorial pcs; diagnosis of periodontitis, stage III with PD ≥ 6 mm and CAL ≥ 5 mm; radiographic loss of alveolar bone. G3: pcs with smoking history (n ≥10 cigarettes per day); diagnosis of periodontitis, stage III with PD ≥ 6 mm and CAL ≥ 5 mm; ra-diographic loss of alveolar bone. EC: reported psychiatric disorders or psychotic medications; systemic diseases; pregnant, lactating, and menopausal; history of taking antibiotics or hormone therapy; acute diseases; immunosuppressive therapy; history of periodontal treatment over the past 6 months	PD, CAL, PI, GI, PBI	Serum and salivary levels of ghrelin and cortisol	Positive association between stress, smoking and stage III periodontitis. In patients with stage III periodontitis, stress and smoking habit increase the severity of destruction of the periodontal tissues. Serum and salivary ghrelin levels were lowest, while serum and salivary cortisol levels were higher in group 3, revealing an inverse relationship between the two parameters.
Yarkac et al., 2018 Turkey [28]	Randomized–controlled trial	60: 30 pregnant women (Pr) 30 non-pregnant women (N-Pr)	F 20–45 N-Pr (27.93 ± 6.61) Pr (28.93 ± 4.04)	IC: presence of gingivitis; PD ≤ 3 mm in all four quadrants; with at least 20 teeth. EC: history of anti-inflammatory, antimicrobial, and hormone therapy in the previous 6 months; systemic diseases; mental disorders; female smokers; breastfeeding; pregnant or menstruating	Clinical and radiographic criteria (clinical criteria for PR only) recorded at baseline time T0 and 3 weeks after nonsurgical periodontal treatment. PD, PI, GI; collection of gingival crevicular fluid (GCF) for evaluation of IL-10 e IL-6.	PSS-10; salivary cortisol (at T0 and T3)	Pregnant women with stress had higher salivary cortisol levels after nonsurgical periodontal treatment than nonpregnant women.
Rajhans et al., 2017 India [23]	Case-control	60-G 1: chronic periodontitis with DM; G 2: chronic periodontitis without DM; G3: systemically and periodontally healthy individuals.	NR 35–50	EC: systemic diseases; pregnant women; history of taking drugs.	PI, GI, PD, CAL	PSS, SRRS	There seems to be a strong correlation between periodontal destruction, DM stress, and serum cortisol levels.
Ramesh et al., 2018 India [24]	Transversal	27 G1: 10 generalized chronic periodontitis G2: 7 generalized aggressive periodontitis G3: 10 periodontally healthy subjects	Both sexes 19–60	EC: periodontal treatment within the previous 3 months; smokers and alcohol users; systemic diseases and history of taking immunosuppressive drugs.	GI, PD, CAL	PHQ-9	Clinical depression could be a risk factor in the development of periodontal disease. No significant association with chronic periodontitis.

### Risk of Bias in Studies

**Figure 2:**
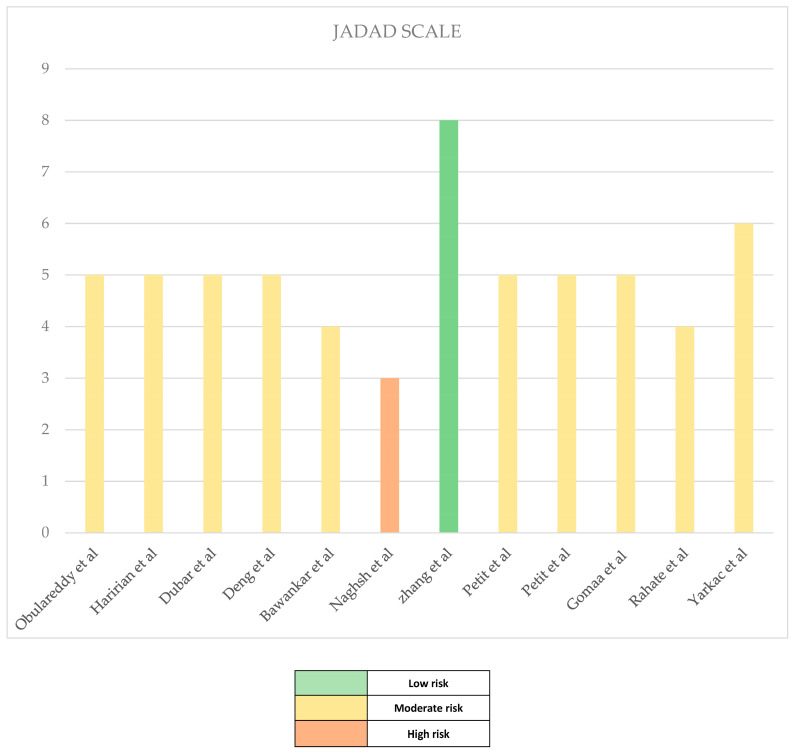
Histogram JADAD scale [19,20,22,28,29,30,31,32,33,35,36,42].

To assess the quality of the studies included in the review and define the most relevant ones, a Quality Assessment table was designed, compiled according to the criteria of the modified JADAD Scale. Using the data, a histogram was developed (Figure 2).

All case–control observational studies and cross-sectional studies were evaluated with a low risk of bias, obtaining a quality assessment score of 6 to 8 points, with most of these studies having adjusted for confounding variables. In addition, appropriate case and control definitions and periodontal examination were provided, and validated tools for the assessment of psychological stress and anxiety were used.

## 4. Discussion

It has been suggested in the literature that adverse socioeconomic conditions elicit a stress response, which can trigger periodontal inflammation. Wellappulli et al. [38], Gomaa et al. [42] limited themselves to determining the association between psychological distress and chronic periodontitis and socioeconomic standing and determining the contribution of psychosocial stress and its hormones in these relationships. The likelihood of having chronic periodontitis was higher in those with psychological distress than in those without psychological distress. In the study by Tanveer et al. [39], it was hypothesized that increased levels of psychological stress among socially disadvantaged women would show a clinically compromising influence on their periodontal health. The association between psychosocial stress and periodontitis among socially disadvantaged women residing in nursing homes was significant.

The goal set by Varshini and Rajasekar [16], Wijayaa et al. [41], Maruyama et al. [26] and Coelho et al. [37] was to determine the effect of stress on periodontal healt, through the administration of questionnaires for the evaluation of depression, anxiety and stress to students at the university faculty of dentistry. The results of that study suggest that students with severe levels of depression, anxiety and stress had greater pocket depth and clinical attachment loss. Therefore, psychological factors have a negative effect on periodontal health. In the study of Varshini and Rajasekar, the instrument does not only measure stress. It suggests that dental students with extremely severe levels of depression, anxiety and stress presented with increased pocket depth and clinical attachment loss. Therefore, psychological factors have an adverse effect on periodontal health.

Several studies presented used saliva samples, cortisol, α-amylase, β-endorphin, chromogranin (CgA), salivary IgA, and various other reactive oxygen metabolites ROM to investigate their relationship to periodontitis. The severity of chronic periodontitis was found to increase, with the increase in cortisol levels and salivary reactive. This therefore indicates that the probability of the onset of periodontitis being greater in subjects with a level of cortisol and another reactive is increased. 

Clinical depression could be a probable risk factor in the development of periodontal disease, especially aggressive periodontitis. As demonstrated by the studies by Ramesh et al. [23] and Folayan et al. [40], mild depression was associated with a higher likelihood of moderate/severe gingivitis in the study population.

The study by Fenol et al. [21], conducted on a total of 70 male inmates, aimed to in-vestigate a possible relationship between psychosocial stress and periodontal disease, demonstrating a significant correlation between clinical parameters, stress and salivary cortisol levels.

Rajhans et al. [23] aimed to correlate the possible stress relationship in patients with chronic periodontitis (CP) and diabetes mellitus (DM), in a sample of 60 individuals aged between 35 and 50 years. All patients underwent psychological assessment using the Perceived Stress Scale (PSS), Social Readaptation Rating Scale (SRRS) and biochemical analysis for serum cortisol estimation. From the results of this study, there seems to be a strong correlation between inflammation of the periodontal tissues, DM stress and serum cortisol levels.

To evaluate the association between the influence of work stress and coping style on periodontitis among Japanese workers, Islam et al. [25] used the Co-Labo57+ self-administered questionnaire and assessed periodontal status based on the inspection vision by dentists, suggesting that low coping style versus high work stress is significantly associated with an increased risk of periodontitis.

In the study performed by Haririan et al. [36], the aim was to compare stress-related neuropeptides in serum and saliva in periodontal health and disease, as well as deter-mine if these markers are related to periodontal parameters and situations of psychological stress. Due to a low response rate to stress questionnaires, this study states that among the different neuropeptides screened, only salivary levels of NPY and VIP were detected in high concentrations in patients with AgP and CP, who could be potential salivary biomarkers for periodontal disease independent of psychological stress.

The primary purpose of the study performed by Deng et al. [32] was to explore the validity and reliability of the hospital anxiety and depression scale (HANS) and the 10-item perceived stress scale (PSS-10) in patients with periodontitis. The secondary aim was to evaluate the psychological characteristics of patients with periodontitis. The latter two parameters, at the end of the study, proved to have good psychometric properties in terms of internal reliability; in this regard, they can be used as general measures for the psychological evaluation in patients with periodontitis. Furthermore, the present study showed a higher prevalence of anxiety, depression and stress in patients with periodontitis.

The aim in the study by Petit et al. [30,31] was to evaluate the influence of psycho-logical stress on the outcomes of non-surgical periodontal treatment in patients of the French population diagnosed with severe chronic periodontitis and with follow-up at six months. Their psychological state was assessed at baseline using self-administered questionnaires, Depression, Anxiety, Stress Scale (DASS) and Toulouse Coping Scale (TCS). At the baseline, before the treatment and six months after the latter, a blood sample was taken to evaluate the plasma levels of cortisol and chromogranin-A and the periodontal indices were recorded. At the end of the study, patients with increased stress, anxiety and depression scores, as well as those using negative coping strategies, showed worse nonsurgical periodontal treatment outcomes.

The cross-sectional study by Rahate et al. [22] and Zhang et al. [33] aimed to study the serum and salivary levels of ghrelin and cortisol and the level of IL-1B in smoking and non-smoking periodontitis patients. In conclusion, the study demonstrated a positive association between stress, smoking and staged periodontitis. Clinical parameters suggest that, in patients with periodontitis, the presence of stress and smoking habits increase the severity of destruction.

Most of the 28 articles included in the study confirm and demonstrate a positive as-sociation between psychological stress and periodontal disease; in detail, two articles examined the positive correlation between stress and gingivitis [28,40], while the remainder demonstrated a relationship between periodontitis and stress.

The only study, of moderate quality, that was unable to detect this association is that of Haririan et al. [36]. While two studies, (Develioglu et al. [27]; Dubar et al. [29]), the first of low quality and the second of moderate quality, found a correlation between the presence of high levels of salivary cortisol in patients with periodontitis, these results did not match those of self-administered questionnaires to patients. Additionally, in the study of Develioglu et al., there was no relationship between STAI 1 and STAI 2 scores and the severity of chronic periodontitis However, since most of the studies have reported positive results, it can be said that psychological stress is a risk factor for periodontitis.

## 5. Conclusions

Numerous studies have shown the mechanisms through which chronic stress negatively affects periodontal tissues. Stress indirectly affects periodontal health through behavioral and lifestyle changes, amplifying the consumption and abuse of smoking and alcohol, poor diet, negligence in oral hygiene and poor compliance with dental care; it would also seem to influence periodontal health through direct biological impact, mediated through saliva alteration, changes in gingival blood circulation and influencing the host immune response. Future studies should focus on the correlation between the pathology and stress and mainly on possible ways of preventing it.

In light of the results obtained from this review, it is important that oral health professionals, also for general health purposes, consider stress factors among the risk factors of periodontal disease, its severity and decreased efficacy of treatments. In patients with chronic stress, it is advisable to carry out brief reminders for periodontal maintenance through constant motivation, strengthening oral hygiene procedures and educating patients on the negative consequences of behaviors used to cope with stress.

## Figures and Tables

**Table 1:** Terms used in the research.

Database	Search Format
PUMBED	(“Stress, Psychological” [Mesh]) AND “Periodontitis” [Mesh]); (“Stress, Psychological” [Mesh]) AND “Periodontal Diseases” [Mesh].
SCOPUS	ALL(“Psychological stress”) AND ALL(“Periodontal Diseases”); ALL(“Psychological stress”) AND ALL(“Periodontitis”); TITLE-ABS-KEY(“Psychological stress”) AND TITLE-ABS-KEY(“Periodontal Diseases”); TITLE-ABS-KEY(“Psychological stress”) AND TITLE-ABS-KEY(“Periodontitis”).
WEB OF SCIENCE	TS = (“Psychological stress”) AND TS = (“Periodontal Diseases”); TS = (“Psychological stress”) AND TS = (“Periodontitis”)

## Data Availability

Further information regarding the data are available upon request to the corresponding author.
